# Impaired myelination and reduced brain ferric iron in the mouse model of mucolipidosis IV

**DOI:** 10.1242/dmm.021154

**Published:** 2015-12-01

**Authors:** Yulia Grishchuk, Karina A. Peña, Jessica Coblentz, Victoria E. King, Daniel M. Humphrey, Shirley L. Wang, Kirill I. Kiselyov, Susan A. Slaugenhaupt

**Affiliations:** 1 Center for Human Genetic Research andDepartment of Neurology, Massachusetts General Hospital and Harvard Medical School, 185 Cambridge Street, Boston, MA 02114, USA; 2 Department of Biological Sciences, University of Pittsburgh, 519 Langley Hall, 4249 Fifth Avenue, Pittsburgh, PA 15260, USA

**Keywords:** Lysosome, Myelination, Oligodendrocytes, Transient receptor potential channel mucolipin-1

## Abstract

Mucolipidosis type IV (MLIV) is a lysosomal storage disease caused by mutations in the *MCOLN1* gene, which encodes the lysosomal transient receptor potential ion channel mucolipin-1 (TRPML1). MLIV causes impaired motor and cognitive development, progressive loss of vision and gastric achlorhydria. How loss of TRPML1 leads to severe psychomotor retardation is currently unknown, and there is no therapy for MLIV. White matter abnormalities and a hypoplastic corpus callosum are the major hallmarks of MLIV brain pathology. Here, we report that loss of TRPML1 in mice results in developmental aberrations of brain myelination as a result of deficient maturation and loss of oligodendrocytes. Defective myelination is evident in *Mcoln1^−/−^* mice at postnatal day 10, an active stage of postnatal myelination in the mouse brain. Expression of mature oligodendrocyte markers is reduced in *Mcoln1^−/−^* mice at postnatal day 10 and remains lower throughout the course of the disease. We observed reduced Perls' staining in *Mcoln1^−/−^* brain, indicating lower levels of ferric iron. Total iron content in unperfused brain is not significantly different between *Mcoln1^−/−^* and wild-type littermate mice, suggesting that the observed maturation delay or loss of oligodendrocytes might be caused by impaired iron handling, rather than by global iron deficiency. Overall, these data emphasize a developmental rather than a degenerative disease course in MLIV, and suggest that there should be a stronger focus on oligodendrocyte maturation and survival to better understand MLIV pathogenesis and aid treatment development.

## Abstract (precis)

**Summary:** Loss-of-function mutations in the *MCOLN1* gene encoding transient receptor potential channel mucolipin-1 (TRPML1) result in a devastating lysosomal storage disorder, mucolipidosis IV. Here, we report that loss of TRPML1 in mice causes developmental aberrations of brain myelination.

## INTRODUCTION

Mucolipidosis type IV (MLIV) was originally described in 1974 based on clinical manifestations that resembled other mucolipidoses, but with a distinct biochemical profile (Berman et al., 1974). In 2000, the gene mutated in MLIV was identified as *MCOLN1*, which encodes the protein mucolipin-1 (Bargal et al., 2000; Bassi et al., 2000; Sun et al., 2000). Based on homology with transient receptor potential (TRP) ion-channel proteins, mucolipin-1 is classified as a founding member of the TRPML family and is also known as TRPML1. Owing to its localization in the lysosomes and the observed lysosomal storage disease phenotype of MLIV, TRPML1 is presumed to regulate membrane traffic, lysosomal biogenesis or lysosomal ion content (Colletti and Kiselyov, 2011; Wang et al., 2014). The diagnostic ultrastructural hallmark of MLIV is the accumulation of storage bodies filled with mixed lamellar and granular electron-dense content, called ‘compound’ bodies (Bargal and Bach, 1989; Berman et al., 1974). The biochemical composition of this storage material includes gangliosides, phospholipids and acidic mucopolysaccharides (Bach et al., 1975, 1977; Bach and Desnick, 1988; Bagdon et al., 1989; Bargal and Bach, 1988, 1989; Slaugenhaupt et al., 1989).

MLIV is primarily found in the Ashkenazi Jewish population (approximately 75% of cases), with a carrier frequency of ∼1:100 (Altarescu et al., 2002; Bach, 2001). There are approximately 100 diagnosed individuals in the USA. Worldwide numbers are largely unknown, but affected individuals with Arab (Mirabelli-Badenier et al., 2014), African-American (Noffke et al., 2001), French Canadian (Berman et al., 1974) and Turkish descent (Tuysuz et al., 2009) have been reported. Over two dozen mutations causing MLIV have been described, the vast majority of which lead to mRNA instability and complete loss of protein (Wakabayashi et al., 2011). A small fraction of MLIV-causing mutations, some of which might be associated with a milder phenotype, lead to aberrant localization and/or ion-channel dysfunction (Chen et al., 2014; Kiselyov et al., 2005). Establishing a genotype-phenotype correlation in MLIV is complicated because of the low incidence of the disease.

MLIV is a developmental disorder that causes motor and cognitive deficiencies, which become noticeable during the first year of life. On average, maximal neurological development in MLIV-affected individuals corresponds to the level of 15-18 months of age and remains stable throughout the third decade of life (Altarescu et al., 2002). In most affected individuals, neurological symptoms include spasticity, hypotonia, an inability to walk independently, ptosis, myopathic facies, drooling, difficulties in chewing and swallowing, and severely impaired fine-motor function (Altarescu et al., 2002). Ophthalmic symptoms are progressive and result in blindness in the second decade of life (Riedel et al., 1985; Smith et al., 2002; Wakabayashi et al., 2011).

Human brain pathology data in MLIV are limited to only two autopsy cases, in which gliosis, abnormal white matter and numerous storage inclusions in all types of brain cells have been reported (Folkerth et al., 1995; Tellez-Nagel et al., 1976). Brain magnetic resonance imaging studies revealed dysgenic corpus callosum, impaired myelination in the white matter and decreased T2 signal intensities in the thalamus as a result of increased ferritin deposition (Frei et al., 1998; Schiffmann et al., 2014).

At present there is no therapy for MLIV, and one of the central factors hindering the search for treatments is a limited understanding of MLIV brain pathology. Mouse models of human disease provide an opportunity to gain a deeper insight into disease pathogenesis and progression. The *Mcoln1* knockout mouse is an excellent model of the human disease, because all hallmarks of MLIV are present in these mice, with the exception of corneal clouding (Venugopal et al., 2007). MLIV mice display brain and gastric phenotypes identical to those reported in humans affected with MLIV (Chandra et al., 2011; Grishchuk et al., 2014). *Mcoln1^−/−^* animals display no immediate phenotype at birth. At 2 months of age, they start to display motor and cognitive deficits that can be detected in the open field test (Grishchuk et al., 2014). Motor deficits in MLIV mice progress to total hindlimb paralysis and death by 8-9 months of age (Venugopal et al., 2007). Post-mortem brain analysis shows lysosomal inclusions in multiple cell types, including neurons, astrocytes, oligodendrocytes, microglia and endothelial cells. Defective myelination and gliosis, the hallmarks of human MLIV, are present in the young adult mice at the onset of cognitive and motor deficits (Grishchuk et al., 2014). However, we observed no neuronal loss in the *Mcoln1^−/−^* forebrain even at the late stage of disease (Grishchuk et al., 2014). These findings prompted investigation of glial cell involvement in MLIV. In the present study, we use the MLIV mouse model to analyze the dynamics of myelination and oligodendrocyte development in the MLIV mouse and to investigate the role of iron dyshomeostasis in MLIV pathogenesis. We show abnormal oligodendrocyte development and reduced ferric iron levels in the white matter of the *Mcoln1^−/−^* mice. These findings highlight the role of TRPML1 in proper brain myelination and suggest that there should be a stronger focus on oligodendrocyte maturation and survival in the search for potential therapeutic approaches in MLIV.

**TRANSLATIONAL IMPACT**

**Clinical issue**

Mucolipidosis type IV (MLIV) is a lysosomal storage disease caused by mutations in *MCOLN1*, which encodes the lysosomal ion channel TRPML1 (mucolipin-1). The disease is characterized by delayed motor and cognitive development, which becomes noticeable during the first year of life. Neurological development in individuals with MLIV corresponds on average to that of an unaffected individual at 15 months of age and remains stable in the second and third decades of life. White-matter abnormalities and a hypoplastic corpus callosum are the major hallmarks of brain pathology in MLIV. At present, however, pathogenesis of MLIV is poorly understood and thus there is no specific treatment for this disease.

**Results**

The authors show that loss of TRPML1 in the mouse model of MLIV (*Mcoln1* knockout mouse) results in reduced myelination in both the white-matter tracts and the gray matter of the brain, similar to what is observed in MLIV human brains. Furthermore, their data indicate that oligodendrocyte differentiation, maturation and region-dependent loss of oligodendrocytes contribute to dysmyelination in *Mcoln1^−/−^* mice. Iron is a key factor required for oligodendrocyte development and myelination, and iron deficiency in children is often associated with hypomyelination and neurological disabilities. The authors observed reduced Perls’ staining in the *Mcoln1^−/−^* brain, indicating lowered levels of ferric iron, although the total iron content in the brain was not changed. These findings indicate that dysmyelination in MLIV could be caused by impaired iron handling in the brain. Mechanistically, the role of TRPML1 in iron trafficking in the brain could be explained either by its function as a lysosomal iron channel that releases ferrous iron from the lysosome to the cytoplasm, or by its involvement in the endolysosomal membrane trafficking pathways that are important for iron delivery and distribution within the brain.

**Implications and future directions**

Iron is known to be vital for brain development, yet mechanisms of its regulation are poorly understood. This study provides evidence that TRPML1 might be an important player, with implications for understanding the molecular basis of MLIV. Future work will be focused on determining the mechanism underlying dysmyelination in MLIV, with the aim of devising new therapeutic strategies for this incurable, devastating disease. Beyond this rare disease, a better understanding of the role of TRPML1 in brain iron regulation provides a new target for therapies aimed at overcoming the consequences of childhood brain iron deficiency, the most common micronutrient deficiency worldwide.

## RESULTS

**Fig. 1.**
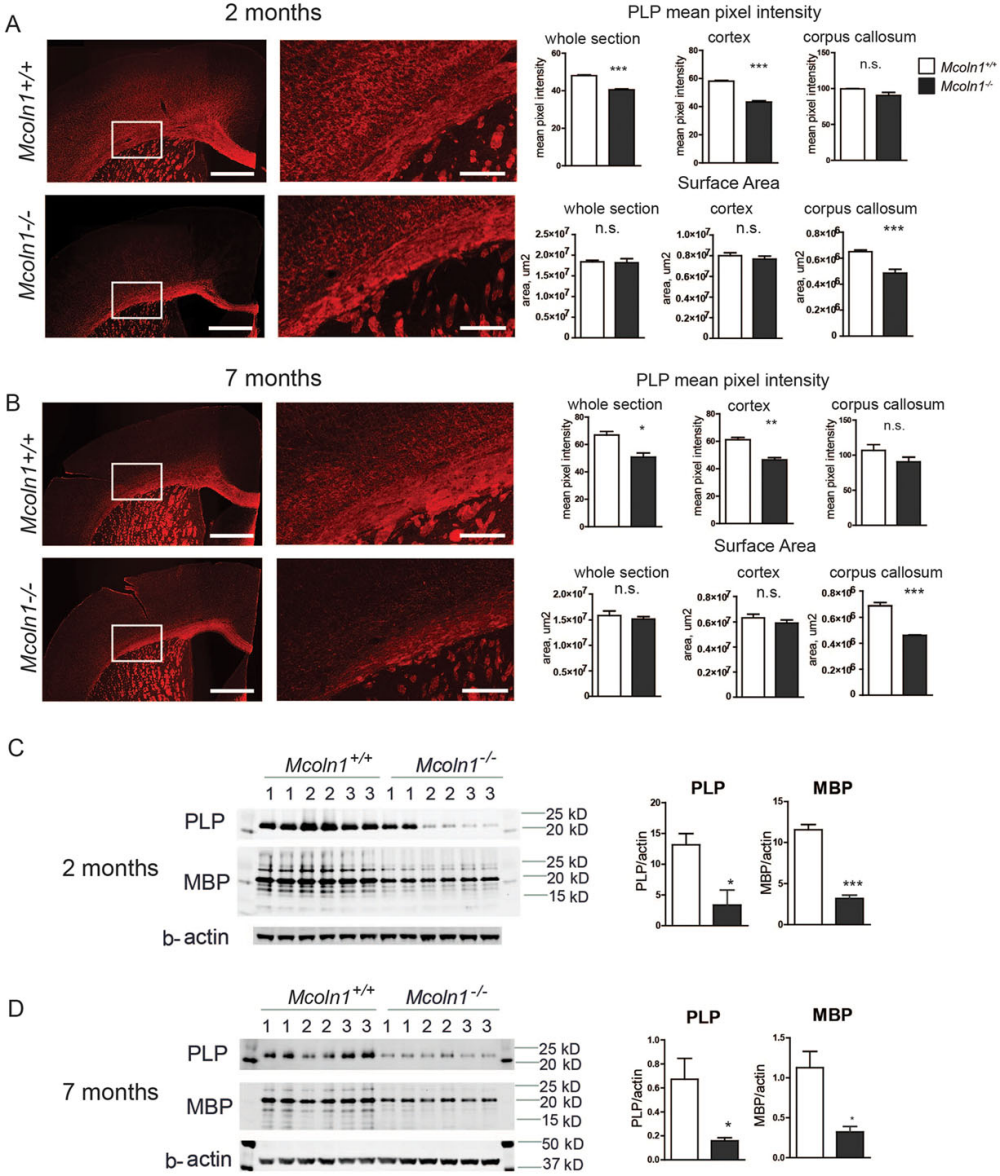
**Reduced myelination in *Mcoln1^−/−^* mouse brain.** (A,B) Proteolipid protein (PLP) immunostaining on coronal brain hemi-sections from 2- (A) and 7-month-old (B) *Mcoln1^−/−^* mice and control littermates shows reduced mean pixel intensity in both the hemi-section and cortical region, and reduced surface area of corpus callosum in *Mcoln1^−/−^* sections. Right image panels represent magnified images of the regions of interest shown in the white boxed areas in the left panels. Scale bars: left panels=500 µm; right panels=100 µm. *n*=5 per genotype (2 months old); *n*=3 per genotype (7 months old). Bar graphs on the right show statistical analysis of mean pixel intensities and surface areas obtained using these sample groups. (C,D) Western blot images and quantification show a significant reduction in the level of PLP and myelin basic protein (MBP) in cerebral cortex homogenates from *Mcoln1^−/−^* mice at the age of 2 (C) and 7 months (D). Samples from three individual mice per genotype group are loaded in duplicates. All graphs represent average values ±s.e.m. Student's *t*-test: *P<0.05, n.s. *P*>0.05.

Immunostaining of brain coronal sections with antibodies against mature myelinating oligodendrocyte marker proteolipid protein (PLP; Baumann and Pham-Dinh, 2001; Han et al., 2013) showed reduced myelination in *Mcoln1^−/−^* mice (Fig. 1). We observed a significant reduction in the PLP immunoreactivity measured in the whole hemispheric coronal section or in the cortical region in adult mice (Fig. 1A,B). We also observed a significant reduction in the surface area of the myelinated corpus callosum. Both findings were present in the early-symptomatic (2 months of age) and the late stage *Mcoln1^−/−^* mice (7 months of age), but were not progressive in the course of disease. Similar data were obtained on the brain sections from 2-month-old *Mcoln1^−/−^* and wild-type littermate mice, immunostained with the antibodies against myelin basic protein (MBP; Fig. S1; Huang et al., 2013). This striking pattern of reduced myelination and thinned corpus callosum is similar to the previously reported FluoroMyelin Green staining performed in *Mcoln1^−/−^* mice at 2 and 7 months of age (Grishchuk et al., 2014). Reduced and non-progressive myelination in *Mcoln1^−/−^* mice was also confirmed by western blotting, which showed a fourfold decrease in the levels of PLP and MBP in the cerebral cortex at the age of 2 and 7 months (Fig. 1C,D). Interestingly, myelination deficits (shown as reduced levels of MBP) were also detected outside the central nervous system, in sciatic nerves of *Mcoln1^−/−^* mice (Fig. S2).

**Fig. 2.**
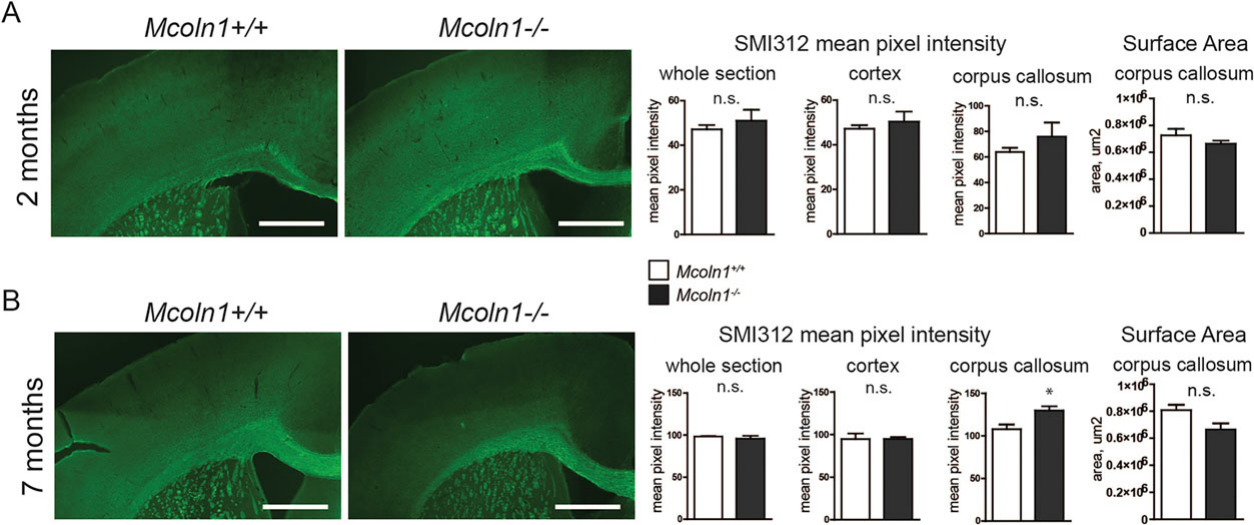
**Preserved axonal projections in *Mcoln1^−/−^* mouse brain.** Immunostaining with the pan-axonal marker SMI312 of coronal brain hemi-sections from 2- (A) and 7-month-old (B) *Mcoln1^−/−^* mice and control littermates shows no significant difference in SMI312 mean pixel intensity or surface area of corpora callosa between control and *Mcoln1^−/−^* sections, except for a slight increase in SMI312 mean pixel intensity in the corpus callosum of 7-month-old *Mcoln1^−/−^* mice. All graphs represent average values ±s.e.m. Student's *t*-test: *P<0.05, n.s. *P*>0.05.

In order to determine whether reduced myelination in the white and gray matter of the *Mcoln1^−/−^* brain is secondary to a lower density of axons, we performed staining with the pan-axonal marker SMI312 (Vasung et al., 2011). Strikingly, we observed no changes in the surface area of the SMI312-stained corpus callosum and in the SMI312 immunoreactivity in the whole hemi-brain sections, cortical region or corpus callosum between genotypes at 2 and 7 months of age (Fig. 2A,B). These data show that white matter abnormalities and reduced gray matter myelination are not the result of a reduced density of axonal fibers in *Mcoln1^−/−^* mice.

**Fig. 3.**
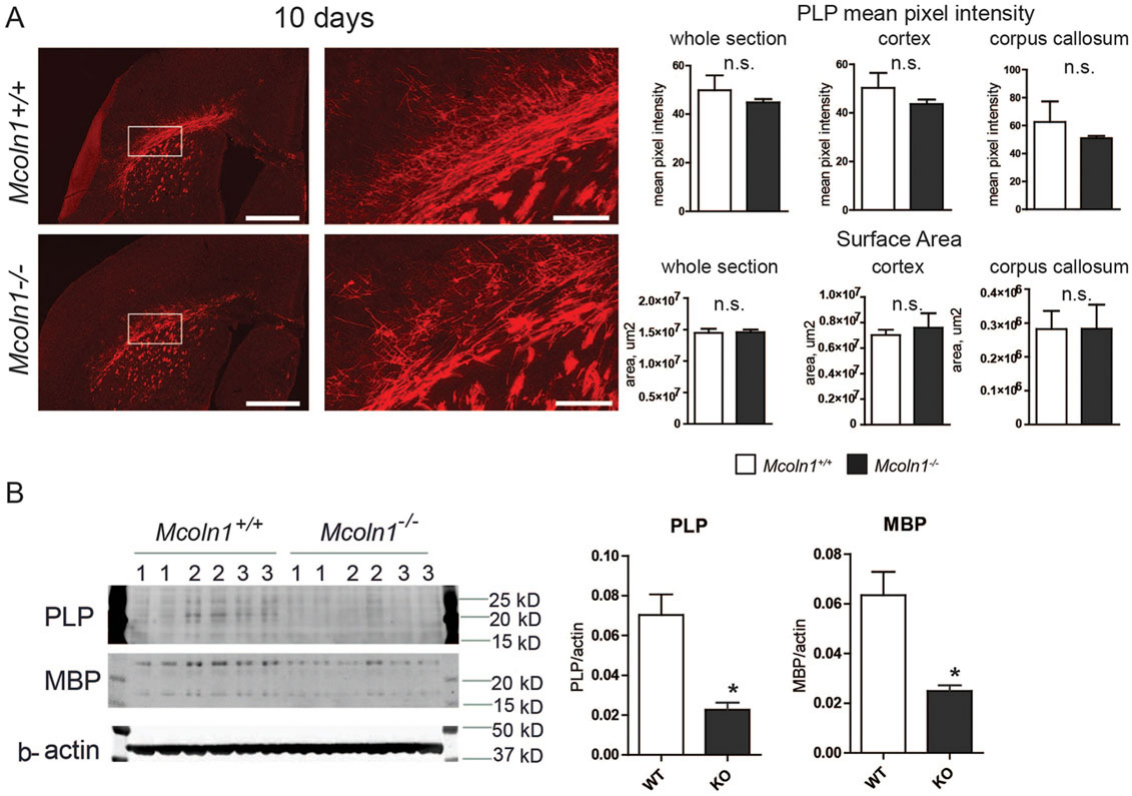
**Myelin disruption in 10-day-old *Mcoln1^−/−^* mice.** (A) Proteolipid protein (PLP) immunostaining on coronal brain hemi-sections shows morphologically altered, ‘patchy’ myelination in *Mcoln1^−/−^* mice compared with wild-type littermates, whereas no significant changes in the overall mean pixel intensity or surface area were observed. Right panels are magnified images of the regions of interest shown in the white boxed areas of the left panels. Scale bars: left panels=500 µm; right panels=100 µm. Bar graphs on the right show statistical analysis of mean pixel intensities and surface areas obtained using these sample groups. (B) Western blot images and quantification showing significant reduction in the levels of PLP and myelin basic protein (MBP) in cerebral cortex of *Mcoln1^−/−^* pups. Samples from three individual mice per genotype group are loaded in duplicates. All graphs represent average values ±s.e.m., *n*=3 per genotype. Student's *t*-test: *P<0.05, n.s. *P*>0.05.

In order to determine whether the loss of TRPML1 alters the developmental stage of myelin deposition, we analyzed myelination markers at postnatal day 10, an active phase of brain myelination in mice. PLP immunostaining of brain sections showed a disorganized pattern of myelinating fibers in the corpus callosum and in the cortex of *Mcoln1^−/−^* pups (Fig. 3A), although the overall intensity was not significantly different between genotypes at this developmental stage. Quantitative analysis of fiber morphology in the corpus callosum showed a significant reduction in the number of branches, number of junctions and number of triple and quadruple points in *Mcoln1^−/−^* pups (Table 1 and Fig. S3), reflecting decelerated development of the myelinated network owing to loss of TRPML1. Moreover, western blot analysis of cortical homogenates showed reduced levels of PLP and MBP in *Mcoln1^−/−^* mice (2.5- and threefold, respectively; Fig. 3B). Overall, these data indicate early developmental changes in brain myelination as a result of loss of TRPML1.

**Table 1.**
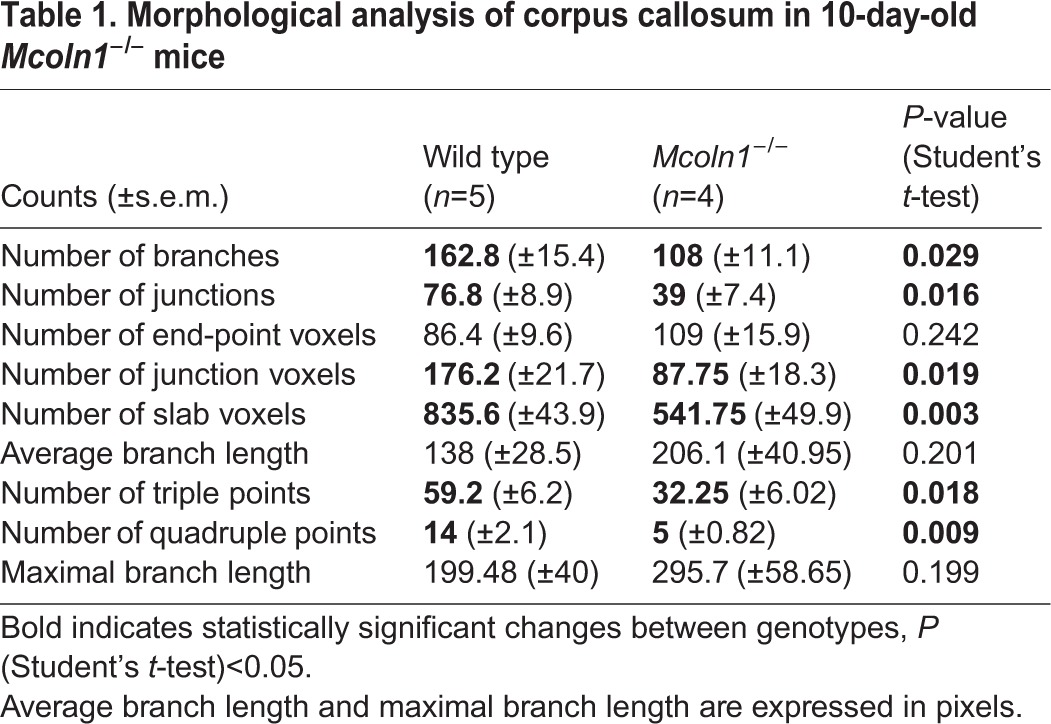
**Morphological analysis of corpus callosum in 10-day-old** ***Mcoln1^−/−^*** **mice**

**Fig. 4.**
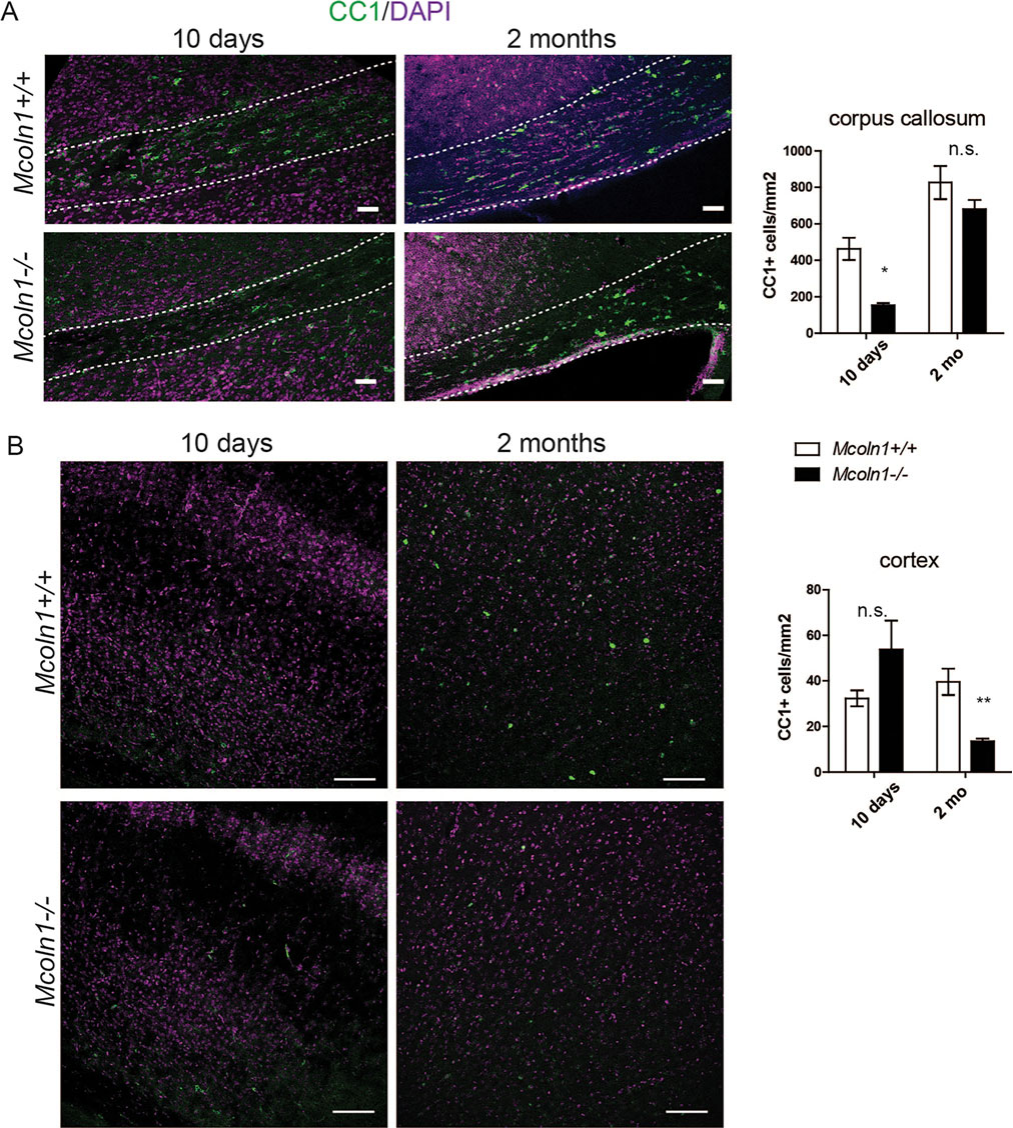
**Decreased density of oligodendrocytes in *Mcoln1^−/−^* brain.** (A) Adenomatous polyposis coli (APC-CC1) immunostaining in the corpus callosum (outlined by white dotted lines) of 10-day- and 2-month-old *Mcoln1^−/−^* mice and their wild-type littermates. Scale bars: 50 µm. Two-way ANOVA of CC1-positive (CC1+) cell (postmitotic oligodendrocyte) density shows a significant effect of age (*F*=47.11; *P*<0.0001) and genotype (*F*=12.34; *P*=0.0034). CC1+ cell density decreased significantly in *Mcoln1^−/−^* corpora callosa at 10 days of age (Bonferroni post hoc test, *P*<0.01). Postnatal day 10, *n*=4 per genotype; 2 months old, *n*=5 per genotype. (B) APC-CC1 immunostaining in cerebral cortex of 10-day- and 2 month-old *Mcoln1^−/−^* mice and their wild-type littermates. Scale bars: 50 µm. Two-way ANOVA of CC1+ cell density shows a significant interaction between age and genotype (*F*=14.74; *P*=0.0024). CC1+ cell density decreased significantly in *Mcoln1^−/−^* cortex at 2 months of age (Bonferroni post hoc test, *P*<0.01). Postnatal day 10, *n*=3 per genotype; 2 months old, *n*=5 per genotype.

The observed reduction of myelination resulting from the loss of TRPML1 could reflect the following factors: (1) the loss of differentiated oligodendrocytes or their abnormal differentiation; (2) deficient myelin formation by *Mcoln1^−/−^* oligodendrocytes; or (3) a combination of both. In order to determine whether reduced myelination in the *Mcoln1^−/−^* brain is caused by reduced numbers of oligodendrocytes, we performed immunostaining with adenomatous polyposis coli (APC-CC1), a marker for postmitotic premyelinating oligodendrocytes, in 10-day-old and 2-month-old mice. Analysis of CC1-positive (CC1+) cell density revealed that the number of oligodendrocytes is significantly reduced in the *Mcoln1^−/−^* corpus callosum at 10 days of age, but the CC1+ cell density improved by 2 months of age (Fig. 4A). Interestingly, in the cortical region the CC1+ cell density was the same at 10 days of age, but a loss of cortical oligodendrocytes was evident by 2 months. The observed difference between the corpus callosum and the cortex suggests that there are region-specific oligodendrocyte responses in the *Mcoln1^−/−^* brain, which could be caused by differences in the microenvironment in these brain structures or by cell-intrinsic differences between white- and gray-matter oligodendrocytes. Although the mechanism is largely unknown, differences in oligodendrocyte differentiation and survival, as well as differences in pathology in multiple sclerosis lesions between white and gray matter have been recently reported (Hill et al., 2014, 2013; Stadelmann et al., 2008).

**Fig. 5.**
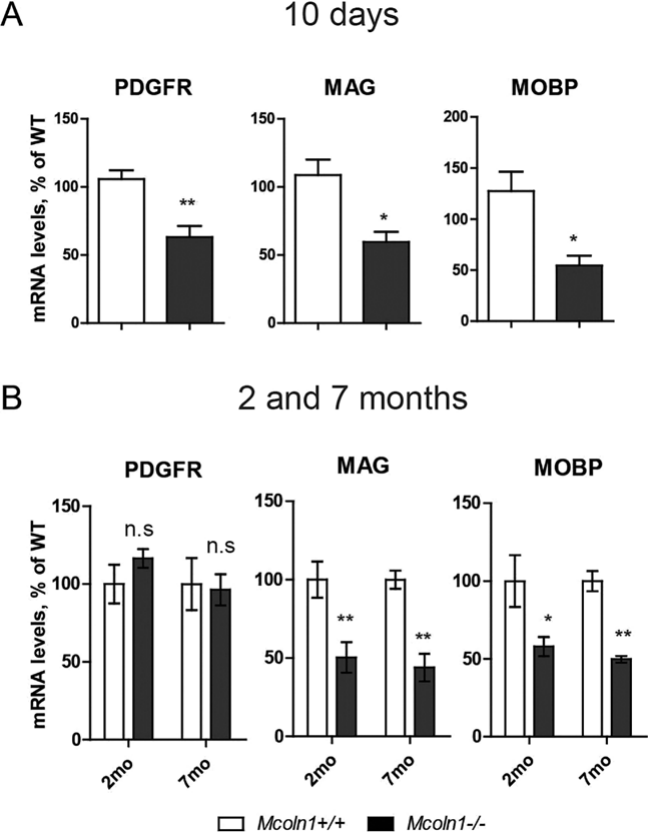
**Decreased markers of oligodendrocyte precursors and mature oligodendrocytes in *Mcoln1^−/−^* mouse brain.** qRT-PCR analysis of *Pdgfra*, *Mag* and *Mobp* expression in 10-day-old (A), 2- and 7-month-old (B) *Mcoln1^−/−^* and wild-type (WT) brains (*n*=4 per genotype for each time point). RNA of each sample was extracted from whole brain. qRT-PCR reactions were performed blind to the investigator using numerically coded samples. Each reaction was performed in duplicate. The data were analyzed using the comparative Δ*Ct* method and presented as a percentage of WT values. Student's *t*-test was used for statistical analysis of postnatal day 10 data; two-way ANOVA with Bonferroni correction was used to assess the effect of genotype and age on mRNA levels for 2- and 7-month-old data. **P<0.01, *P<0.05, n.s. *P*>0.05.

Next, we performed qRT-PCR gene-expression analysis using whole-brain RNA extracts from 10-day-old, 2- and 7-month-old mice. We observed reduced levels of mRNA encoding the mature oligodendrocyte markers myelin-associated glycoprotein (MAG) and myelin-associated oligodendrocyte basic protein (MOBP; Han et al., 2013) in *Mcoln1^−/−^* mice at 10 days (Fig. 5A). Significant decline in mRNA levels of *Mag*  and *Mobp* persisted in *Mcoln1^−/−^* mice at 2 and 7 months of age (Fig. 5B), but was not progressive in the course of disease as shown by a significant effect of genotype (*Mag*, *F*_genotype_=32.96, *P*<0.0001; *Mobp*, *F*_genotype_=23.67, *P*=0.0004) and no significant effect of age (*Mag*, *F*_age_=0.12, *P*=0.73; *Mobp*, *F*_age_=0.19, *P*=0.67) by two-way ANOVA. These data provide further confirmation that deficient myelination in *Mcoln1^−/−^* brain occurs during development and persists throughout the course of disease.

In order to determine whether impaired differentiation of oligodendrocyte precursors contributes to a decreased density of postmitotic oligodendrocytes and hypomyelination in the *Mcoln1^−/−^* brain, we analyzed expression of the oligodendrocyte precursor marker PDGFRα (Han et al., 2013; Perez et al., 2013) in *Mcoln1^−/−^* and wild-type mice at 10 days, 2 and 7 months of age. We observed a significant reduction in *Pdgfra* mRNA levels in 10-day-old *Mcoln1^−/−^* mice, and no significant differences between genotypes in adult mice when myelination is largely complete (Fig. 5A,B). Lower levels of *Pdgfra* transcripts at 10 days of age might indicate a reduced number of oligodendrocyte precursors in *Mcoln1^−/−^* brain at this critical stage of myelination, perhaps limiting their differentiation, maturation and myelin deposition. Restoration of the *Pdgfra* mRNA levels in adult *Mcoln1* knockout mice is not accompanied by restoration of the mature oligodendrocyte or myelin markers, highlighting the importance of the time-dependent regulation of this process and perhaps multiple roles of TRPML1 in the oligodendrocyte cell lineage.

**Fig. 6.**
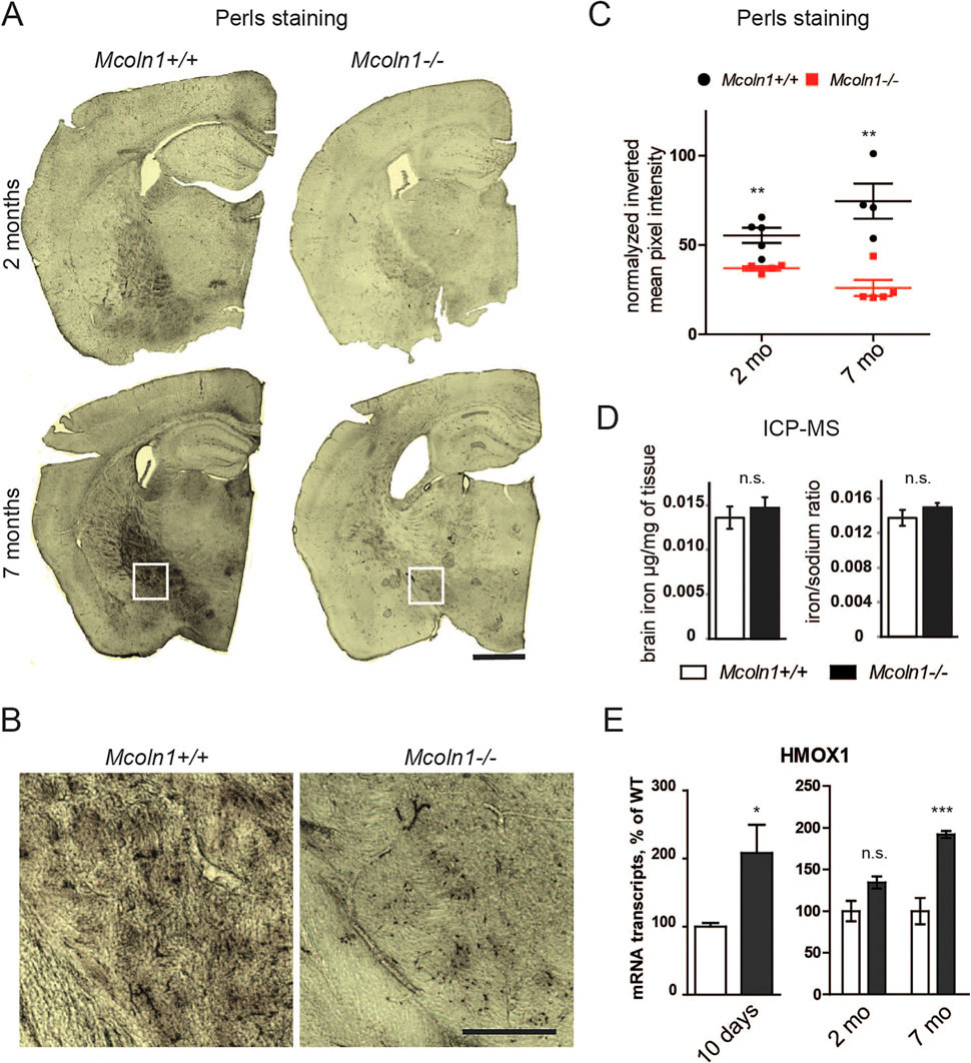
**Decreased levels of ferric iron and oxidative stress in the *Mcoln1*^−/−^ brain.** (A) Coronal brain sections stained for Fe^3+^ content by modified Perls' protocol. Note an increase of iron deposits with age in the white matter of control mice and reduced staining in the *Mcoln1*^−/−^ brain sections. Scale bar: 1 mm. (B) Higher-magnification images of Perls'-stained wild-type (WT) and *Mcoln1^−/−^* brain sections from white boxed areas shown in (A) demonstrate lower staining density in *Mcoln1^−/−^* white matter. Scale bar: 50 µm. (C) Quantification of Perls' staining shows significantly reduced staining in *Mcoln1^−/−^* brains at both 2 and 7 months of age (Student's *t*-test: *P*=0.025 and *P*=0.02, respectively); WT, *n*=5; *Mcoln1^−/−^*, *n*=4 at 2 months, and WT, *n*=4 *Mcoln1^−/−^*, *n*=5 per genotype at 7 months. (D) Inductively coupled plasma mass spectrometry (ICP-MS) analysis of brain cortex homogenates of 7-month-old *Mcoln1^+/+^* and *Mcoln1^−/−^* mice done in a blind experiment (*n*=4 per genotype). The differences are not significant (*P*=0.52 and *P*=0.30). (E) Oxidative stress in mucolipidosis type IV brain. qRT-PCR analysis of *Hmox1* expression in whole-brain homogenates of *Mcoln1^+/+^* and *Mcoln1^−/−^* mice (*n*=4 per genotype). *Actb* was used as a housekeeping gene. The experiments were performed blind to investigator. The data are presented as a percentage of WT mRNA levels ±s.e.m. Student's *t*-test was used for comparison between genotypes for data obtained in 10-day-old mice; *P<0.05. Two-way ANOVA of data sets at 2 and 7 months showed a significant increase of *Hmox1* expression with age (*F*_genotype×age_=7.2; *P*=0.014); Bonferroni post hoc test: ***P<0.0001, n.s. *P*>0.05.

Given that iron is an important factor for oligodendrocyte differentiation and maturation (Todorich et al., 2009) and that TRPML1 is directly involved in intracellular iron trafficking (Coblentz et al., 2014; Dong et al., 2008), we set out to test the iron status in the *Mcoln1^−/−^* brain. We stained brain sections with Perls' stain, which detects ferric iron bound to ferritin (Meguro et al., 2005). As the most iron-rich cells in the brain, mature oligodendrocytes are frequently visualized using this technique (Schonberg and McTigue, 2009). *Mcoln1^+/+^* brains contained clearly identifiable Perls' stain, but such staining was significantly reduced in white-matter tracts in *Mcoln1^−/−^* brains, indicating a loss of H-ferritin-bound iron (Fig. 6A-C). Measurement of total iron content (Fe^2+^ and Fe^3+^) in the unperfused brain by inductively coupled plasma mass spectrometry (ICP-MS) showed no statistically significant differences in total iron levels between *Mcoln1^−/−^* and wild-type mice, which rules out systemic iron deficiency in *Mcoln1^−/−^* mice (Fig. 6D). These data show that loss of *Mcoln1* results in iron dyshomeostasis in the brain, which is likely to play a role in the observed impairment of brain myelination in MLIV.

Iron dyshomeostasis is known to lead to oxidative stress in the brain (Abeliovich and Flint Beal, 2006; Chen et al., 2013; Hare et al., 2013; Schrag et al., 2011). Therefore, we evaluated the levels of the marker *Hmox1* mRNA in the brain of *Mcoln1^−/−^* and wild-type mice. Importantly, *Hmox1* mRNA was significantly elevated in *Mcoln1^−/−^* brain at all ages evaluated, indicating prolonged oxidative stress (Fig. 6E).

Overall, our data suggest that myelination deficits in *Mcoln1^−/−^* mice are caused by defects in the formation of the myelin sheath, rather than by axonal loss. Our demonstration that markers of oligodendrocyte precursors, premyelinating and mature myelinating oligodendrocytes are all disrupted in *Mcoln1^−/−^* mouse brain suggests that loss of TRPML1 affects all stages of oligodendrocyte development. Furthermore, our results show impaired iron handling in the brain as a result of loss of TRPML1. Given that iron is an important trophic factor for oligodendrocyte differentiation and maturation, improper iron handling as a result of loss of TRPML1 might account for the observed dysmyelination in mucolipidosis IV. Our results, although descriptive in nature, provide the first evidence of impaired iron handling and early myelination defects in MLIV, highlight the developmental nature of mucolipidosis IV and emphasize the rationale for a stronger focus on oligodendroglia and early-stage myelination as potential targets for the development of therapeutics.

## DISCUSSION

In the present study, we show that hypomyelination in the MLIV mouse brain is associated with decreased expression of both precursor and mature oligodendrocyte markers, as well as a decrease in the number of postmitotic oligodendrocytes. Our observation of delayed myelin deposition during early postnatal brain development highlights the developmental character of brain pathology in MLIV. Interestingly, our data also indicate that the fate of developing oligodendrocytes is affected differently in the white (corpus callosum) versus gray (neocortex) matter of the brain as indicated by changes in CC+ cell counts (Fig. 4) or PLP and MBP staining intensities in these brain regions. This suggests that the tissue microenvironment and factors released by neighboring cells can contribute to the ability of *Mcoln1^−/−^* oligodendrocytes to develop, survive and/or produce myelin. Despite these region-specific changes within the brain, our data demonstrate that TRPML1 is an important regulator of myelination, both in the central nervous system and in peripheral nerves. Given the absence of overt neuronal loss in MLIV (Grishchuk et al., 2014), combined with the preservation of axonal fibers (Fig. 2) in the *Mcoln1^−/−^* brain, oligodendrocyte development and survival should be high-priority targets for the development of therapeutics.

The main function of oligodendrocytes is myelin synthesis and the formation of myelin sheaths around axons to facilitate action potential propagation. Pediatric disorders of myelin, such as periventricular white matter injury and hereditary leukodystrophies, result in cerebral palsy-like and cognitive dysfunction similar to those documented in MLIV (Pouwels et al., 2014). Proliferation of oligodendrocyte precursors, differentiation and maturation relies on several trophic factors, including FGF, PDGF, IGF-1, thyroid hormone and iron (Morath and Mayer-Proschel, 2001; Rouault, 2013; Todorich et al., 2009). Oligodendrocytes are the most iron-rich cells in the brain. As the most metabolically active cells in the brain, oligodendrocytes require massive amounts of ATP, and iron is involved in oxidative phosphorylation. Iron is a cofactor for a number of enzymes directly or indirectly involved in myelin synthesis, including lipid saturase and desaturase (Nakamura and Nara, 2004; Rao et al., 1983; Todorich et al., 2009). Furthermore, iron deficiency causes a delay in oligodendrocyte maturation and hypomyelination in both humans and rodents (Hare et al., 2013; Todorich et al., 2009). Injection of ferritin-bound iron into white matter leads to an increase in the levels of oligodendrocyte progenitor and mature oligodendrocyte markers (Schonberg et al., 2012; Schonberg and McTigue, 2009). An increase in intracellular ferritin levels is also evident during the oligodendrocyte maturation process (Li et al., 2013).

TRPML1 is clearly required for oligodendrocyte development and function, and this is perhaps what underlies the observed brain pathology in MLIV. The precise role of TRPML1 in oligodendrocyte development is currently unknown; however, several studies highlight possibilities and suggest a link to the role of TRPML1 in iron handling. How might mucolipin-1 regulate iron handling? One possibility is through its function in endolysosomal trafficking. Mucolipin-1 localizes to late endosomes and lysosomes (Kiselyov et al., 2005; Miedel et al., 2006; Vergarajauregui and Puertollano, 2006) and is known to regulate endolysosomal fusion and membrane trafficking (Dong et al., 2010; LaPlante et al., 2004; Pryor et al., 2006; Samie et al., 2013; Thompson et al., 2007; Vergarajauregui et al., 2008). In most tissues, iron is absorbed by endocytosis of Fe^3+^-transferrin or Fe^3+^-ferritin (Valerio, 2007). In the endolysosomal compartment, Fe^3+^ is liberated from the protein complex, converted to Fe^2+^ and absorbed into the cytoplasm via endolysosomal membranes. In the cytoplasm, Fe^2+^ is oxidized to Fe^3+^ and binds to cytoplasmic ferritin. From the cytoplasm, iron enters mitochondria for energy production, binds to transferrin for trafficking or exporting, serves as a cofactor for enzymatic reactions, such as myelin production, or binds to cytoplasmic ferritin and other scavengers for storage. Hence, based on the role of mucolipin-1 in endocytic membrane trafficking, it might regulate Fe^3+^-ferritin delivery to the lysosomes.

The second possibility is based on the permeability of TRPML1 to divalent heavy metal ions, including Fe^2+^ (Dong et al., 2008), so it could facilitate the direct transport of Fe^2+^ from lysosome to cytoplasm. The common transferrin-dependent iron entry pathway relies on the divalent transporter DMT1 (Siah et al., 2006) for iron release from endolysosomes into the cytosol. Interestingly, oligodendrocytes depend strictly on extracellular ferritin as a source of iron (Hulet et al., 2000; Todorich et al., 2009, 2011, 2008), and the role of DMT1 in iron handling specifically in oligodendrocytes has been disputed (Burdo et al., 2001; Song et al., 2007). Thus, owing to its Fe^2+^ permeability, mucolipin-1 might supplant DMT1 in oligodendrocytes totally, or during some stages of development, thus making oligodendrocytes especially vulnerable to the loss of mucolipin-1. Our demonstration of reduced levels of ferritin-bound ferric iron in *Mcoln1^−/−^* brain by Perls' stain provides experimental support for this idea. Iron bound to ferritin, specifically to H-ferritin, is prevalent in oligodendrocytes and can be visualized using Perls' stain. Reduced Perls' staining in white-matter tracts of *Mcoln1^−/−^* mice indicates loss of biologically available, H-ferritin-bound iron. Given that other brain cell types, including neurons, rely on transferrin endocytosis and DMT1 for iron delivery (Hare et al., 2013; Moos and Morgan, 2004; Paz Soldan and Pirko, 2012; Perez et al., 2013; Todorich et al., 2011; Valerio, 2007), it is possible that loss of mucolipin-1 might not affect neuronal iron handling or total iron content. Supporting this hypothesis, our ICP-MS data show no changes between *Mcoln1^−/−^* and control mice in the total amount of iron in the unperfused brain tissue. Therefore, the maturation or myelination deficits in MLIV cannot be explained by a general brain iron deficiency. Rather, they are directly related to oligodendrocyte malfunction.

Given that TRPML1 is permeable to Fe^2+^ but not to Fe^3+^, it is possible that loss of TRPML1 leads to lysosomal build-up of Fe^2+^, which is known to catalyze Fenton-like reactions that result in the formation of reactive oxygen species. Previously, oxidative stress has been shown both in *in vitro* cellular models (Coblentz et al., 2014) and in a *Drosophila* model (Venkatachalam et al., 2008), and in the present study we show, for the first time, evidence of oxidative stress *in vivo* in the MLIV mouse brain.

Regardless of the precise role of the ion channel TRPML1 in regulating iron in the brain, our studies suggest that it is a new target for pharmacological intervention for conditions linked to brain iron handling and myelination. Our data also indicate that restoration of myelin is an attractive therapeutic strategy for MLIV, and further studies will reveal whether mechanisms leading to impaired myelination in MLIV rely solely on oligodendrocyte intrinsic (cell-autonomous) mechanisms.

## MATERIALS AND METHODS

### Animals

*Mcoln1* knockout mice were maintained and genotyped as previously described (Venugopal et al., 2007). The *Mcoln1^+/−^* breeders for this study were obtained by backcrossing onto a C57Bl6J background for more than 10 generations. *Mcoln1^+/+^* littermates were used as controls. Experiments were performed according to the institutional and US National Institutes of Health guidelines and approved by the Massachusetts General Hospital Institutional Animal Care and Use Committee.

### Immunohistochemistry and image analysis

To obtain brain tissue for histological examination, 10-day-old, 2- and 7-month-old *Mcoln1^−/−^* and control mice were transcardially perfused under isoflurane anesthesia with ice-cold PBS followed by 4% paraformaldehyde in PBS. Brains were postfixed in 4% paraformaldehyde in PBS for 24 h, washed with PBS, cryoprotected in 30% sucrose in PBS overnight, frozen in isopentane and stored at −80°C. Brains were bisected along the midline, and one hemisphere was examined histologically. Coronal sections 40 μm thick were cut using a Microm freezing microtome and collected into 96-well plates containing TBSAF (TBS, 30% ethylene glycol, 15% sucrose and 0.05% sodium azide). These sections were stored at 4°C prior to any staining procedures. For experiments, the corresponding serial sections were used for each brain. For PLP, MBP and SMI312 staining, the sections were microwaved in citrate buffer (10 mM citric acid and 0.05% Tween 20, pH 6.0) at low power for 10 min for antigen retrieval, blocked in 0.5% Triton X-100 and 5% normal goat serum (NGS) in PBS and incubated with primary antibodies diluted in 1% NGS overnight. The following primary antibodies were used: PLP rabbit polyclonal, 1:500, ab28486; MBP (SMI99), mouse monoclonal, 1:1000, ab24568; SMI312 (pan axonal neurofilament) mouse monoclonal 1:1000, ab24574; all from Abcam (Cambridge, MA, USA). Sections were incubated with secondary antibodies in 1% NGS for 1.5 h at room temperature. The following secondary antibodies were used: goat anti-mouse AlexaFluor 488 and donkey anti-rabbit AlexaFluor 555 (1:500; Invitrogen, Eugene, OR, USA). For APC-CC1 staining, no antigen retrieval was performed. After blocking, the sections were incubated with anti-APC-CC1 antibody (mouse monoclonal, 1:100, OP80; Calbiochem, Billerica, MA, USA), followed by goat anti-mouse AlexaFluor 633 (1:500; Invitrogen, Eugene, OR, USA). Sections were counterstained with NucBlue nuclear stain (Life Technologies, Eugene, OR, USA) and mounted on microscopic slides.

For PLP, MBP and SMI312 staining, images for analysis were acquired on a Nikon 80i upright epifluorescence microscope with Hamamatsu Orca CCD camera (Nikon, Tokyo, Japan) with 5× objective and NIS-Elements 4.2 software (Nikon, Tokyo, Japan) using an automated stitching function. The exposure time was set to avoid saturation and was the same for all sections (wild-type and *Mcoln1^−/−^* sections within the same immunohistochemistry experiment). Image analysis was performed using FIJI software (NIH, Bethesda, MD, USA). Areas of interest (whole hemi-section, cortical region or corpus callosum) were selected in each section. Area and mean pixel intensity values were compared between genotypes using GraphPad Prizm 5 software (GraphPad, La Jolla, CA, USA) using Student's *t*-test.

Quantitative morphological analysis of corpus callosum was done using the AnalyzeSkeleton plugin of ImageJ (Arganda-Carreras et al., 2010). Regions of interests (ROI; 200 pixels ×200 pixels) of corpus callosum were selected on images of PLP-stained sections of 10-day-old brains. Selections were converted to 8-bit images, made binary and skeletonized using the Skeletonize 2D/3D option and analyzed using the AnalyzeSkeleton plugin. An investigator was blinded to genotypes. Total counts of number of branches, junctions, end-point voxels, junction voxels, slab voxels, triple and quadruple points, average and maximal branch lengths per ROI per mouse were averaged across animals in genotype groups (*n*=5 WT and *n*=4 *Mcoln1^−/−^*) and compared in GraphPad Prizm 5 software using Student's *t*-test.

For APC-CC1 analysis, we acquired images of two non-overlapping fields of view in somatosensory or motor cortex and corpus callosum using a 20× objective (HCX PL APO CS 20.0×0.70 DRY UV) and confocal laser scanning microscope Leica TCS SP5 (Leica Microsystems Inc., Wetzlar, Germany). The number of CC1+ cells was counted in FIJI in corresponding areas of interest (cortex or corpus callosum). CC1 cell density was expressed as the number of cells per square millimeter of the tissue. Statistical analysis of data (two-way ANOVA with Bonferroni correction to assess the effect of genotype and age) was performed using GraphPad Prizm 5.

### Perls’ staining

For Perls' staining, the sections were incubated in potassium ferrocyanide with HCl [10% potassium ferrocyanide:10% HCl (2:1)] for 60 min with rocking. Sections were washed with water, incubated with inactivated 3,3′-diaminobenzidine (DAB) (no H_2_O_2_ added) and activated DAB, for 30 min each time. After washing in water, sections were mounted on microscope slides, dried and coverslipped. Bright-field images were acquired using a Leica DMI6000 microscope using DFC425 camera and HCX PL S-APO 5.0×0.15 objective (Leica Microsystems Inc.). Inverted mean pixel intensities were measured in FIJI. For background normalization, inverted mean pixel intensity measured outside the brain tissue was subtracted from inverted mean pixel intensity values for every image. Student's *t*-test was used for comparisons between genotypes.

### Western blot analysis

For immunoblotting, fresh-frozen pieces of sensory-motor cortex were homogenized in the lysis buffer (20 mmol/l HEPES, pH 7.4, 10 mmol/l NaCl, 3 mmol/l MgCl_2_, 2.5 mmol/l EGTA, 0.1 mmol/l dithiothreitol, 50 mmol/l NaF, 1 mmol/l Na_3_VO_4_ and 1% Triton X-100; reagents from Sigma, LaJolla, CA, USA) and a protease inhibitor cocktail (catalog number 11873580001; Roche, Mannheim, Germany). Homogenates were centrifuged at 2100 ***g*** for 10 min at 4°C, supernatants collected and protein concentration was determined using Pierce BSA Protein Assay kit (ThermoFisher Scientific, Waltham, MA, USA). Sciatic nerves were homogenized in radioimmunoprecipitation assay buffer (RIPA; Boston BioProducts, Ashland, MA, USA) supplemented with 0.1 mmol/l EDTA and a protease inhibitor cocktail (catalog number 11873580001; Roche, Mannheim, Germany) according to Vargas et al. (2010). Proteins (20 μg for cortex and sciatic nerve homogenates from 2- and 7-month-old mice; 40 μg for 10-day-old cortex homogenates) were separated by SDS-PAGE on a 4-12% Bis-Tris gel 3-(*N*-morpholino)propansulfonic acid (MOPS) buffer (Invitrogen, Waltham, MA, USA) and transferred onto nitrocellulose membrane (ThermoFisher Scientific). The following primary antibodies were used: anti-proteolipid protein (ab28486, 1:1000, rabbit polyclonal; Abcam, Cambridge, MA, USA), anti-myelin basic protein (NE1019, mouse monoclonal, 1:1000; EMD Millipore, Billerica, MA, USA), anti-β-actin (A5441, 1:3000, mouse monoclonal; Sigma). After incubation with primary antibody, the following secondary antibodies were applied: polyclonal goat anti-mouse or goat anti-rabbit IgG conjugated with IRDye 680 or IRDye 800, both from LI-COR (Lincoln, NE, USA). Protein bands were visualized using the Odyssey Infrared Imaging System and analyzed by Odyssey v3.0 software (LI-COR). In densitometric analysis, optical density values were normalized by β-actin. All data were expressed as mean±s.e.m. Data were analyzed statistically using Student's *t*-test.

### RNA extraction and qRT-PCR

For qRT-PCR experiments, total RNA was extracted from brain tissue from 10-day-old, 2- and 7-month-old *Mcoln1^−/−^* and control mice using TRizol (Invitrogen, Carlsbad, CA, USA), according to the manufacturer's protocol. For complementary DNA synthesis, MuLV Reverse Transcriptase (Applied Biosystems, Foster City, CA, USA) was used with 2 μg of total RNA and oligo(dT)18 (IDT, Cralville, IA, USA) as the primer. qRT-PCR was carried out using 1:75 dilutions of complementary DNA, 2× SYBR Green/ROX Master Mix (Fermentas, Glen Burnie, MD, USA), and 4 μM primer mix per 10 μl reaction. For gene-expression analysis, the following primers were used (IDT): *Pdgfrα*, forward 5′-TGGAAGCTTGGGGCTTACTTT-3′ and reverse 5′-TAGCTCCTGAGACCTTCTCCT-3′; *Plp*, forward 5′-GTTCCAGAGGCCAACATCAA-3′ and reverse 5′-CCACAAACTTGTCGGGATGT-3′; *Mbp*, forward 5′-GTGCCACATGTACAAGGACT-3′ and reverse 5′-TGGGTTTTCATCTTGGGTCC-3′; *Mag*, forward 5′-CGGGATTGTCACTGAGAGC-3′ and reverse 5′-AGGTCCAGTTCTGGGGATTC-3′; *Mobp*, forward 5′-CACCCTTCACCTTCCTCAAC-3′ and reverse 5′-TTCTGGTAAAAGCAACCGCT-3′; *Hmox1*, forward 5′-CACAGATGGCGTCACTTCCGTC-3′ and reverse 5′-GTGAGGACCCACTGGGAGGAG-3′; and *Actb*, forward 5′-GCTCCGGCATGTGCAAAG-3′ and reverse 5′-CATCACACCCTGGTGCCTA-3′. To avoid amplification of genomic DNA and ensure amplification of complementary DNA, all primers were designed to span exons, and negative RT reactions (without reverse transcriptase) were performed as controls. Samples were amplified in triplicates on the 7300 Real Time System (Applied Biosystems) using the following program: 2 min at 50°C, 10 min at 95°C, and 40 cycles at 95°C for 15 s followed by 1 min at 60°C. In addition, a dissociation curve step was run to corroborate that amplification with specific primers resulted in one product only (Fig. S4). The ΔΔ*Ct* method was used to calculate relative gene expression, where *Ct* corresponds to the cycle threshold. Δ*Ct* values were calculated as the difference between *Ct* values from the target gene and the housekeeping gene *Actb* (Table S1). Experiments were performed as a double blind using numerically coded samples; the sample identity was disclosed at the final stage of data analysis. Data are represented as fold change.

### ICP-MS

ICP-MS analysis of mouse cortical tissues was performed at the Department of Geology and Planetary Science, University of Pittsburgh. Frozen brain samples were coded and analyzed in a blinded manner. Mouse cortical tissues were weighed, dissolved in pure nitric acid, dried, reconstituted in 2% nitric acid and analyzed using a PerkinElmer NexION 350 ICM-MS Spectrometer (PerkinElmer Inc., Waltham, MA, USA). Iron concentration was normalized to the total wet tissue weight. In addition to iron, sodium and other metals were analyzed. As an additional analysis tool, iron concentration was normalized to sodium content.
